# Prostate cancer induced bone pain: pathobiology, current treatments and pain responses from recent clinical trials

**DOI:** 10.1007/s12672-022-00569-z

**Published:** 2022-10-18

**Authors:** A. E. Smith, A. Muralidharan, M. T. Smith

**Affiliations:** 1 St Vincent’s Hospital, Darlinghurst, Sydney, NSW Australia; 2 Neurobiology of Chronic Pain, The Charles Perkins Centre, Faculty of Science, The University of Sydney, Sydney, NSW 2006 Australia; 3 Centre for Integrated Preclinical Drug Development, School of Biomedical Sciences, Faculty of Medicine, The University of Queensland, St Lucia Campus, Brisbane, QLD 4072 Australia

**Keywords:** Prostate cancer induced bone pain (PCIBP), Metastatic castrate-resistant prostate cancer (mCRPC), Analgesics, NSAIDs, Opioids, Morphine, Fentanyl, Ectopic nerve fibre sprouting, Central sensitization

## Abstract

**Purpose:**

Metastatic spread of prostate cancer to the skeleton may result in debilitating bone pain. In this review, we address mechanisms underpinning the pathobiology of metastatic prostate cancer induced bone pain (PCIBP) that include sensitization and sprouting of primary afferent sensory nerve fibres in bone. We also review current treatments and pain responses evoked by various treatment modalities in clinical trials in this patient population.

**Methods:**

We reviewed the literature using PubMed to identify research on the pathobiology of PCIBP. Additionally, we reviewed clinical trials of various treatment modalities in patients with PCIBP with pain response outcomes published in the past 7 years.

**Results:**

Recent clinical trials show that radionuclides, given either alone or in combination with chemotherapy, evoked favourable pain responses in many patients and a single fraction of local external beam radiation therapy was as effective as multiple fractions. However, treatment with chemotherapy, small molecule inhibitors and/or immunotherapy agents, produced variable pain responses but pain response was the primary endpoint in only one of these trials. Additionally, there were no published trials of potentially novel analgesic agents in patients with PCIBP.

**Conclusion:**

There is a knowledge gap for clinical trials of chemotherapy, small molecule inhibitors and/or immunotherapy in patients with PCIBP where pain response is the primary endpoint. Also, there are no novel analgesic agents on the horizon for the relief of PCIBP and this is an area of large unmet medical need that warrants concerted research attention.

## Introduction

For patients with prostate cancer, bone is the most common organ of metastasis with a prevalence of ~ 90% for men with metastatic castrate-resistant disease [19, 80, 99] and a median survival time of 12–53 months [82]. Metastatic spread of prostate cancer to the skeleton is most often located in the vertebrae (69%) followed by the pelvic bones (41%), long bones (25%) and the skull (14%) [106]. These bone metastases may lead to debilitating bone pain and skeletal-related events (SREs) that not only impair patients’ quality of life (QoL) [4, 82] but are also associated with high healthcare costs and a large socioeconomic burden [82]. SREs include pathological skeletal fracture, spinal cord compression, systemic hypercalcemia and anemia [4]. Of these, spinal cord compression is a medical emergency and may result it permanent paralysis, loss of limb function and mobility, and severe pain [96]. In this review, we address the pathobiology of prostate cancer-induced bone pain (PCIBP), current treatment strategies as well as pain responses from clinical trials published in the past seven years (January 2015 to March 2022) [72].

## Prostate cancer metastasis to bone

The exact mechanisms underpinning prostate cancer metastasis to bone in preference to other body sites are unclear. One aspect is that the prostate is highly vascularized with the prostatic venous plexus draining into the internal iliac vein, which connects to the vertebral venous plexus throughout the spinal column [9, 80]. The highly vascular trabecular structure of bone provides an ideal environment for metastatic prostate cancer cells to colonize, including ready access to oxygen and other nutrients [60, 80]. The bone marrow is particularly favoured, due to its slower blood flow, high vascularization, and cell composition including osteoclasts and osteoblasts that control bone remodelling [107]. Increased expression of the integrin, αVβ^3^, on the surface of metastatic prostate tumour cells promotes their adherence to endothelial cells in the bone marrow [93] and expression of receptor activator of nuclear factor kappa-B ligand (RANKL) by prostate cancer cells, promotes their dissemination and colonization of bone [22]. Also, the immunosuppressive tumour microenvironment shields cancer cells from immune surveillance and antitumour activity [4, 80, 86].

In prostate cancer, only rare, phenotypically distinct prostate cancer tumour-initiating cells, also called stem-like prostate cancer cells, have the capacity to form new tumours [59]. However, the mechanisms leading to bone metastasis formation are not fully elucidated [70]. In patients, high expression levels of cyclin A1 and aromatase proteins in metastatic bone lesions support the notion that stem cell-like prostate cancer cells overexpressing cyclin A1 and aromatase, preferentially metastasize to bone [70]. Indeed, cyclin A1 and aromatase increased local production of bone marrow-releasing factors including androgen receptor, Hairy/enhancer-of-split related with YRPW motif-like protein (HeyL), oestrogen, oestrogen receptor alpha, and matrix metalloproteinase 9 (MMP9), that together facilitate metastatic homing to the bone marrow [51, 70, 90]. Thus, local production of steroid hormones and MMPs in the bone marrow likely contribute to generation of a microenvironment suitable for stem-like prostate cancer cells to establish metastatic lesions in bone [51, 70]. The bone microenvironment may also regulate metastatic prostate cancer cells between dormant and proliferative states with dormancy potentially lasting for many months if not years [80, 106].

### Pathobiological bone remodelling in metastatic prostate cancer

Skeletal bone remodelling requires a tight balance between osteoclast-mediated bone resorption and osteoblast mediated bone formation and dysregulation of this balance leads to pathology [38]. At the sites of bone metastases, tumour cells attack the bone and they interact with osteoclasts, osteoblasts, stromal cells and inflammatory cells [109]. Although prostate cancer-induced bone metastasis is primarily osteoblastic in nature, dysregulation of osteoclasts also causes osteolytic lesions [4], which together form osteosclerotic lesions. Prostate tumour cells hyper-secrete endothelin-1 (ET-1), that regulates osteoblast function and contributes potently to PCIBP [4]. Although ET-1 signalling via the endothelin A receptor, stimulates osteoblast proliferation and formation of new bone, this bone is weak and prone to fracture [72, 106]. Activated osteoblasts release RANKL (receptor activator of nuclear factor κB ligand) that interacts with its cognate receptor, RANK, to stimulate proliferation and maturation of osteoclasts which triggers their damaging osteolytic effect on bones [80, 106]. This knowledge led to the development of denosumab (human monoclonal antibody against RANKL) to decrease osteoclastic activity [82]. Although denosumab and bisphosphonates reduce the painful complications of bone metastases, they do not improve overall survival [33].

Osteoclasts produce acidosis-causing protons and adenosine-triphosphate which activate acid-sensing ion channel 3 and the P2 purinoceptor 3 respectively, that are expressed on sensory nerve fibres in bones [103]. Osteoclasts secrete collagenases and proteases that demineralize bone and damage bone matrix protein [106]. Osteoclasts also secrete transforming growth factor-beta and insulin-like growth factor that promote tumour cell proliferation by inhibiting apoptosis [106]. The net outcome is a ‘vicious cycle’ that promotes growth of bone metastases and development of metastatic PCIBP [10].

Within the tumour microenvironment, prostate cancer cells and infiltrating immune cells release an array of pro-inflammatory cytokines including interleukin-1β (IL-1β), IL-6, and tumour necrosis factor alpha (TNFα), as well as chemokines (C–C motif ligands) such as CXCL1, CXCL8 (IL-8), CXCL12 and CCL2, that have a pivotal role in the initiation, progression and metastasis of prostate cancer [58, 71, 100]. However, clinical studies that used monoclonal antibodies to inhibit their pro-tumorigenic effects have been disappointing. For example, addition of the anti-IL-6 monoclonal Ab (mAb), siltuximab (CNT0238) to mitoxantrone/prednisone, reduced expression of IL-6-regulated genes and suppressed known IL-6 signalling pathways including JAK-STAT3 and ERK1/2, but there was no improvement in outcomes for patients with mCRPC [34]. Despite promising efficacy of a CCL2 mAb in rodent models of prostate cancer, sustained suppression of serum CCL2 concentrations was not achieved in patients with mCRPC [83]. A randomized phase Ib/II study (MAGIC-8; NCT03689699) evaluating the effects of nivolumab with or without the IL-8 mAb (BMS-986253), which signals via the chemokine receptors, CXCR1 and CXCR2 [100], in combination with a short course of androgen deprivation therapy in men with castration-sensitive prostate cancer, is currently under investigation. Other ongoing early phase clinical trials in men with mCRPC include the phase 1/2 ACE trial of the small molecule slowly reversible inhibitor of CXCR2, AZD5069, to assess whether its addition to enzalutamide (ADT) will reverse resistance to enzalutamide alone (NCT03177187) [100]. However, blockade of IL-8 signalling via CXCR2 may be insufficient as IL-8 will continue to signal via CXCR1 that is also expressed on prostate cancer cells, stromal and immune cells within the mCRPC microenvironment [100]. To overcome this potential issue, the orally bioavailable small molecule, SX-682, has been developed as a potent allosteric inhibitor of both CXCR1 and CXCR2 [100]. SX-682 is in clinical trials as an add-on treatment to pembrolizumab in patients with metastatic melanoma (NCT03161431), but it may also have applicability in patients with mCRPC [100].

### Sensory and autonomic nerve fibres in bone

**Fig. 1:**
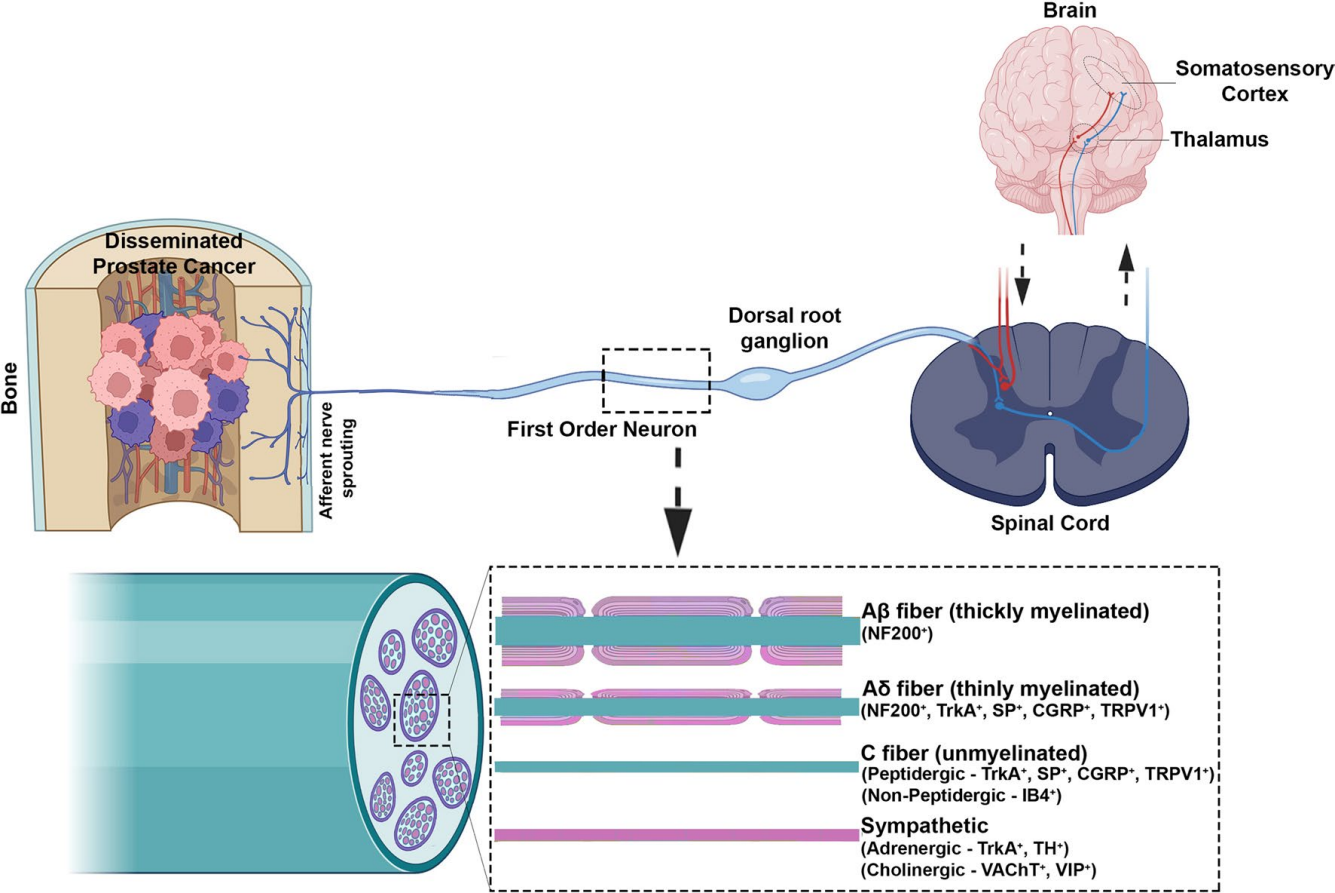
Primary afferent sensory nerve fibres primarily in the periosteum but also in the bone marrow of prostate cancer invaded bone undergo pathobiological sprouting and sensitization with pronociceptive signalling transduced into the dorsal horn of the spinal cord, from where it projects to the brain where it may be interpreted as pain (Created with Biorender.com)

Primary afferent sensory nerve fibres and sympathetic nerve fibres are abundant in the three major bone compartments (periosteum, marrow, mineralized bone). The layer of connective tissue that envelops the bone (i.e., periosteum), provides a supportive microenvironment for vasculature, nerves, and periosteal cells [53]. For sensory nerve fibres, the periosteum is the most densely innervated, with fewer sensory fibres in the mineralized bone and the bone marrow (Fig. 1; Table 1) [4, 15, 106].

**Table 1:** Density of sensory and sympathetic nerve fibres in the distal femur of the mouse, normalized to tissue area (fibres per mm^2^) in the three major bone compartments [15]

Nerve Fibre Density	Periosteum	Marrow	Mineralized bone
CGRP + sensory fibres	177.4	9.9	15.9
NF200 + fibres	154.1	3.3	7.6
TH + fibres	128.4	45.3	60.2

CGRP = calcitonin gene related peptide, a marker of small-diameter unmyelinated sensory nerve fibres; NF200 = neurofilament 200, a marker of medium to large diameter myelinated sensory nerve fibres; TH = tyrosine hydroxylase, a marker of sympathetic nerve fibres

When expressed by fibre number normalized to tissue area in the distal femur of the mouse, sensory axon innervation in the periosteum was dense (Table 1) with a 15- to 25-fold lower abundance in mineralized bone and the bone marrow (Table 1) [15]. In the latter, unmyelinated calcitonin-gene-related-peptide (CGRP) positive sensory afferents were branched and projected to linear, varicose-rich endings, particularly near the epiphyseal trabecular bone, and less frequently in the metaphysis and diaphysis [15]. Myelinated neurofilament 200-positive sensory axons that mostly expressed CGRP, had a similar pattern of distribution but with a relatively longer and more linear morphology [15]. In bone, there are abundant sympathetic adrenergic neurons that express tyrosine hydroxylase (TH) together with cholinergic neurones containing acetylcholine and vasoactive intestinal peptide [15]. In the bone marrow, larger-diameter blood vessels are often enveloped by sympathetic TH-positive fibres rich in varicosities, and sympathetic axons (like sensory fibres) can dissociate from vasculature to terminate as free-nerve endings in the marrow [15]. Sympathetic nerve fibre density was greatest in the periosteum but less (40%) than that for primary afferent sensory nerve fibres (Table 1) [15]. However, the density of sympathetic fibres in mineralized bone and the bone marrow was considerably higher than that of sensory nerve fibres in the corresponding bone compartments (Table 1) [15].

### Pathobiological bone remodelling and sensitization of nociceptors

The pathobiology of PCIBP is underpinned by neuroplastic changes at multiple levels of the somatosensory system and involves complex cascades of interactions between the metastatic tumour cells, host immune cells, and stromal and tumour-associated factors in the bone microenvironment [108]. The infiltration and proliferation of tumour cells in bone alters the phenotype and function of bone-forming osteoblasts and bone-resorbing osteoclasts, resulting in pathological bone remodelling [15]. This further modulates the expression of various osteoclastogenic and osteoblastogenic factors, such as RANKL, prostaglandin E2 (PGE2), parathyroid hormone-related protein (PTHrP), cytokines (e.g., IL-1, IL-6, IL-8, IL-11, TNF-α), macrophage inflammatory protein 1 α (MIP-1α), endothelin (ET)-1, insulin growth factor 1 (IGF-1), wingless-type protein (Wnt), but to name a few [15, 72, 106, 108]. Of interest, many of these factors are pro-inflammatory mediators and well-known to directly sensitize primary sensory afferent fibres (nociceptors) [15, 55, 72].

The periosteum and bone marrow of an adult bone are uniquely innervated largely by tropomyosin kinase A (TrkA^+^) thinly myelinated Aδ-fibres and TrkA^+^ unmyelinated C-fibres, and receive very little innervation by large diameter Aβ fibres and TrkA^−^ C-fibres [49, 55]. These primary afferent fibres express an array of receptors and ion channels, including endothelin A receptors, prostaglandin, TrkA, bradykinin, cytokine, chemokine and purinergic receptors, the transient receptor potential channel, vanilloid subfamily member 1 (TRPV1), and acid-sensing ion channel 3 (ASIC3) (Fig. 1;[4, 44, 55]. The activation of these targets by their cognate ligands from the “pro-inflammatory” soup comprising mediators released from bone stromal cells, osteoclast- and tumour-induced acidosis, and tumour-associated immune cells results in sensitization of nociceptors and transmission of noxious signals to other components of the somatosensory nervous system [55, 72, 103] The intrinsic molecular mechanisms of nociceptor activation by the aforementioned factors are reviewed in detail elsewhere [103, 108].

In addition to sensitization of nociceptors (inflammatory component), seminal works by Mantyh and colleagues have implicated a neuropathic component in the pathobiology of PCIBP. Specifically, studies have shown active and pathological sprouting and neuroma formation by sensory and sympathetic nerve fibres in the periosteum and bone marrow of the metastatic tumour-invaded bone in rodent models of bone cancer pain [14, 49]. This pathological sprouting of nerve fibres was attributed to the actions of nerve growth factor (NGF), and sustained inhibition of NGF, either via administration of anti-NGF or a Pan-Trk inhibitor, markedly attenuated nerve sprouting, neuroma formation and bone pain even in advanced stages of bone cancer [36, 57]. Additionally, administration of anti-NGF (mAb911) has also been shown to suppress functional connectivity alterations in a rodent model of bone cancer pain [20]. However, findings from randomized-controlled clinical trials revealed significant adverse effects of the humanized monoclonal NGF-antibody (tanezumab), including joint damage and rapidly progressive osteoarthritis, resulting in imposition of clinical hold by the Food and Drug Administration on all NGF inhibitors [1].

**Fig. 2:**
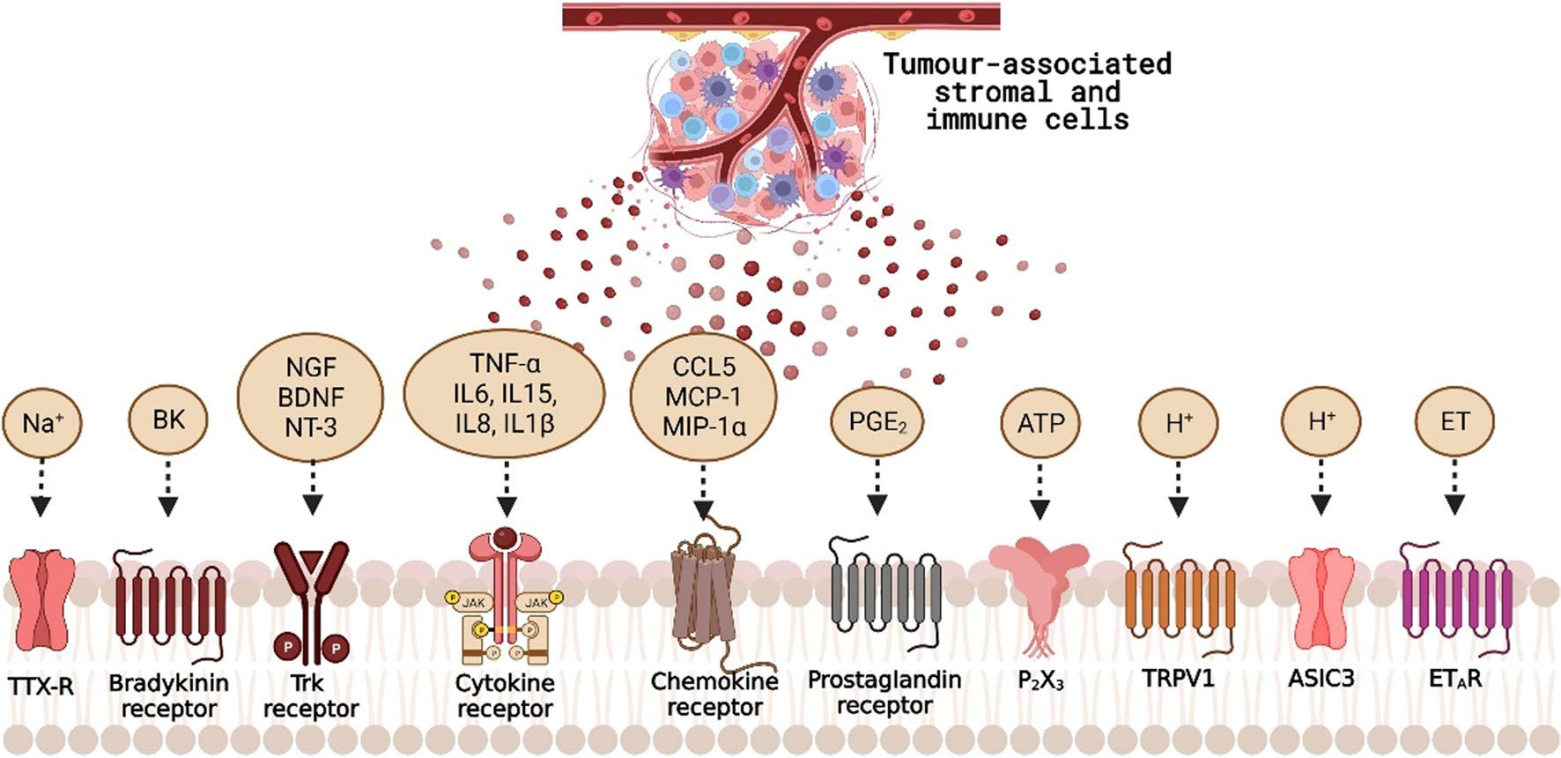
Targets (receptors and ion channels) activated by pronociceptive mediators in metastatic tumour-invaded bone (Adapted from Zajaczkowska et al. 2019 and redrawn with Biorender.com)

The ongoing barrage of pronociceptive input mediated by sensory nerve fibres in prostate cancer-invaded bone induces neuroplastic changes (sensitization) in the cell bodies located in the dorsal root ganglia [102]. Augmented release of pronociceptive neurotransmitters at the central terminals in the dorsal horn of the spinal cord, induces multiple changes in the spinal cord to induce a state of central sensitization [98]. This is important as the dorsal horn is a key centre for relaying pronociceptive information from the cancer-invaded bones to 2nd order neurons that project from the spinal cord to higher centres in the brain where this information may be interpreted as pain (Fig. 2) [108].

### Clinical characteristics of bone pain in patients with metastatic prostate cancer

In prostate cancer, the osteosclerotic nature of bone metastases results in the formation of poor-quality bones and as a result SREs are very common [24]. SREs may cause pain, impair physical activity and negatively impact patients’ QoL [99]. Although the best approach for treating metastatic PCIBP is to improve control of the skeletal disease burden [79], this is not always possible. Also, some patients with a single lesion report severe pain whereas others with disseminated bone lesions experience low to moderate pain [103, 106]. Hence, patients need individualized analgesic/ adjuvant medication treatment regimens for alleviation of PCIBP. Of men who die from prostate cancer, > 90% have bone metastases present [29, 84]

PCIBP presents as intermittent dull aches initially, but as metastatic disease progresses, the bone pain becomes constant and more severe with greater intensity during movement and increased severity at night that is not necessarily relieved by lying down [54, 106]. Pain upon palpation is often present in the vicinity of metastatic bone lesions [106]. Ongoing tumour growth within the bone usually leads to breakthrough (episodic) pain, defined as recurrent episodes of extreme pain breaking through the analgesic dosing regimen used to alleviate the background pain [65, 68] Breakthrough pain has a prevalence of ~ 75% in patients with bone metastases, and it is typically acute, piercing and very severe [65, 68]. Breakthrough pain may occur spontaneously without an obvious trigger, or it may be induced by movement and body weight-bearing (incidental) [65, 68]. Breakthrough pain is unpredictable with a short time to maximum pain intensity (< 5 min), and its often-severe intensity has a major negative impact on the ability of patients to undertake activities of daily living and on their QoL [27, 68].

### Summary

Metastasis of prostate cancer to bone dysregulates the normal balance between osteoclasts and osteoblasts in healthy bone remodelling. The net effect is formation of osteosclerotic lesions comprised of poor-quality bone prone to fracture. Additionally, primary afferent sensory nerve fibres innervating the periosteum and the bone marrow of cancer-invaded bones undergo ectopic sprouting. These fibers are then sensitized by a broad array of pronociceptive mediators present in the inflammatory milieu of the cancer-invaded bone. These mediators interact with their cognate receptors and ion channels to transduce pronociceptive signalling into the dorsal horn of the spinal cord and from there to higher centres in the brain where it may be interpreted as pain. PCIBP initially presents as dull aches but as disease progresses, the bone pain becomes constant and more severe in intensity with it worst upon movement and at night. Patients may also experience rapid-onset breakthrough pain that may be spontaneous or triggered by movement and body weight-bearing. Poorly relieved bone pain has a major negative affect on patient’s QoL. Management of bone pain is addressed in the next section of this review.

## Clinically used analgesic/adjuvant agents for relief of PCIBP

**Fig. 3:**
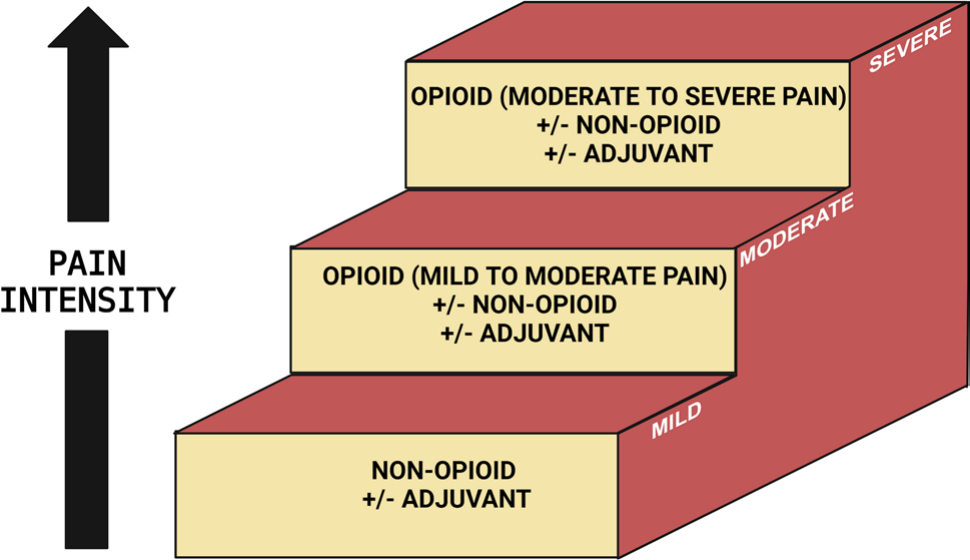
World Health Organisation 3-step analgesic ladder for the management of cancer-related pain [76]. (Created with Biorender.com)

The management of bone metastases in patients with prostate cancer is challenging in terms of morbidity, debilitating pain, impaired function, and poor QoL [45]. Unfortunately, since 2015, there have been no new analgesic agents approved for the relief of PCIBP and so medications succinctly summarized by the World Health Organisation’s 3-Step Analgesic Ladder ([75];Fig. 3), are the mainstay of analgesic therapy [30]. In brief, analgesics for treating background PCIBP include paracetamol and nonsteroidal anti-inflammatory drugs (e.g. ibuprofen and coxibs) for mild pain, with weak opioids such as tramadol or codeine added when pain is mild to moderate in intensity. Weak opioids are replaced by strong opioid analgesics such as morphine when pain is moderate to severe in intensity [30]. Analgesic agents are given orally around the clock [30, 75]. The subcutaneous (s.c.) route is the first-choice alternate route for patients unable to receive opioids by the oral or transdermal routes [30]. Intravenous (i.v.) dosing is an option for opioid titration when rapid pain control is needed and i.v. infusion should be considered when s.c. dosing is contraindicated [30]. For patients with chronic kidney disease with glomerular filtration rates < 30 mL/min), fentanyl and buprenorphine are the safest opioids [30]. When initiating oral morphine therapy for moderate to severe cancer-related pain, it is recommended that patients be individually dose-titrated with immediate-release formulations given every 4 h plus rescue doses (up to hourly) with the daily titrated dose used to convert to a slow-release morphine formulation. The regular dose of slow-release opioids can be adjusted to include the total amount of rescue morphine [30]. Analgesic adjuvant agents such as tricyclic antidepressants (e.g. amitriptyline at doses ≤ 75 mg/kg), duloxetine, and anticonvulsants (e.g. gabapentin, pregabalin) may be added to alleviate a neuropathic component [30, 75]. There is a lack of evidence to support the routine use of the N-methyl-D-aspartate receptor antagonist, ketamine in cancer-related neuropathic pain [30].

For patients with ‘end of dose failure’, the European Society for Medical Oncology (ESMO) guidelines recommend an increase in the dose of strong opioid unless side-effects develop [30]. If the latter, a switch in the dosing route or rotation of the opioid to an equianalgesic dose of a 2nd opioid may be beneficial, and rescue doses are used to treat breakthrough pain [30]. If pain persists despite escalating doses of a strong opioid, it is important to re-assess the pain and treatment, and consider invasive interventions [30]. For the approximately 10% of patients where their cancer pain is difficult to manage with oral or parenteral analgesic agents, interventional techniques include nerve blocks, neurolytic blocks and intrathecal (i.t.) drug delivery [30]. The i.t. route of opioid administration enables logarithmic dose reductions as the opioid is delivered adjacent to the target opioid receptors in the dorsal horn of spinal cord resulting in fewer systemic side-effects [30]. Life expectancy > 6 months justifies the use of an implantable i.t. pump, but only after a trial using a temporary spinal catheter or bolus dose of opioid and local anaesthetic [30].

### Management of opioid-related adverse effects in patients with cancer-related pain

Opioid analgesics evoke a broad array of opioid-related adverse effects with constipation being particularly problematic as tolerance does not develop to constipation. Thus, laxatives must be routinely prescribed for both the prophylaxis and the management of opioid-induced constipation (OIC). The use of naloxone (in association with oxycodone) or methylnaltrexone for managing OIC may be considered [30]. Similarly, naloxegol may be used to treat OIC [27, 76] Anti-dopaminergic drugs and metoclopramide are recommended for the treatment of opioid-related nausea and vomiting [30]. In the event of opioid-induced respiratory depression, naloxone must be used promptly [30].

### Barriers to treatment with opioids

Barriers to appropriate opioid treatment of moderate to severe cancer-related pain such as metastatic PCIBP include fear and apprehension by patients and clinicians [105]. Widespread reporting by the general media of the ‘opioid crisis’, particularly in the United States, may induce opioid phobia in patients leading them to under-report their pain and worsen their suffering [105]. This has led to increased legislative and regulatory activity aimed at tighter controls with respect to opioid prescribing [74]. An unintended consequence may be hesitancy by clinicians to increase opioid dosages for patients with PCIBP due to concerns on addiction, respiratory depression, or decreased life expectancy [105]. To address this issue, the American Society of Clinical Oncology (ASCO) released a policy statement on opioid therapy aimed at protecting access to opioid treatment for cancer-related pain [74]. In particular, patients with cancer and cancer survivors should not be subject to arbitrary prescription limits that artificially limit access to medically necessary treatment with opioids and patients need education on the safe storage of opioid analgesics [74]. Patients may also experience opioid stigma and so interventions targeted to patients, clinicians, and healthcare systems, are needed to de-stigmatize opioid prescription in patients with pain due to advanced cancer such as metastatic prostate cancer [21].

### Rapid-onset opioid analgesic treatments for breakthrough bone pain

Traditionally, breakthrough cancer pain is defined as pain of peak intensity and short duration that occurs in patients who otherwise have stable and acceptable analgesia provided by analgesics given around the clock [61, 65, 68, 69]. The ESMO guidelines recommend oral immediate-release morphine for the relief of breakthrough pain [30], Additionally, fast-onset strong opioid analgesics such as the rapid-onset fentanyl products, are recommended for treating severe breakthrough pain [48, 68]. Their superiority over oral opioids regarding effectiveness and rapid onset of action has been demonstrated in multiple studies [48, 68]. However, their use is restricted to patients who are already receiving, and are tolerant to, opioid analgesics prescribed for managing their background PCIBP to avoid life-threatening respiratory depression [18, 48]. Rapid-onset fentanyl formulations include oral transmucosal products (Table 2) and intranasal fentanyl sprays [7, 27, 37]. Fentanyl is a 100-fold more potent than morphine, it is 90% unionized at physiological pH, and it has high lipophilicity which makes it ideal for oral transmucosal delivery [27]. However, the differential formulation properties and pharmacokinetics of each oral transmucosal fentanyl product (Table 2) mean that they must not be used interchangeably, and each patient must be dose-titrated to an ‘optimal/ successful’ dose to achieve analgesia [18]. The lowest strength dose of each product (Table 2) is recommended to start dose-titration in patients receiving at least 60 mg of oral morphine equivalents (OME) for background pain, which is equivalent to the 3^rd^ step of the WHO Analgesic Ladder [68].

**Table 2:** Comparison of three transmucosal fentanyl products used clinically for relief of breakthrough PCIBP

Transmucosal Fentanyl Products	Fentanyl citrate sublingual tablet (Abstral^™^)	Fentanyl citrate lozenge (Actiq^™^)	Fentanyl citrate buccal tablet (Fentora^™^)	References
Product type	Sublingual non-effervescent orally disintegrating tablet	Lozenge with integral applicator	Disintegrating Buccal tablet	
Product usage	Place on oral mucosa beneath tongue at deepest part. It dissolves in saliva in ~ 1–2 min. Not to be chewed, sucked or swallowed. Allow to completely dissolve in sublingual cavity Fentanyl is absorbed across oral mucosa. Doses must be titrated	Rub lozenge attached to applicator on inside of cheek and move around mouth. Lozenge dissolves in ~ 15 min and not be chewed. If breakthrough pain is relieved before complete dissolution, remove lozenge from mouth Fentanyl is absorbed across oral mucosa. Doses must be titrated	Place on buccal mucosa above 3rd molar & expose to moisture. Effervescent reaction disintegrates tablet in ~ 14–25 min to release fentanyl Not to be sucked, chewed, or swallowed. Blister not to be opened until ready to use Doses must be titrated	[27, 65]
Product technology	Comprises coarse water-soluble carrier particles (granulated mannitol) partially covered by fine dry particles (< 5 μm diameter) of fentanyl citrate. A bioadhesive component (cross-linked polyvinylpyrrolidone) is also added to carrier particles. Fine drug particles adhere to surface of carrier particles by adhesion forces and are optimally exposed to dissolving fluid	Candied matrix technology that contains ~ 2 g of sugar per lozenge	Uses the Oravescent^®^ Technology drug delivery system where transient pH changes accompany an effervescent reaction resulting in both an ↑ in rate of dissolution of ionized drug and ↑rate of absorption of non-ionized drug No sugar in buccal tablet	[16, 32]
Dosages/strengths available (as fentanyl base)	100, 200, 300, 400, 600, 800 μg	200, 400, 600, 800, 1200, 1600 μg	100, 200, 400, 600, 800 μg	[27, 65]
Bioavailability	~ 54%	~ 50%	~ 65%	[27, 65]
Onset of clinically meaningful pain relief	~ 10–15 min	~ 10–15 min	~ 15–30 min	[27, 65]
Time to peak analgesia	60 min	30–60 min	60–90 min	[27]

The mechanism(s) underpinning breakthrough pain have been postulated to differ from those transducing background pain that is adequately controlled with strong opioids [41]. To gain insight on this notion, a female rat model of cancer-induced breakthrough pain induced by unilateral intra-tibial injection of mammary adenocarcinoma cells, was developed [41]. In these animals, movement-associated breakthrough pain induced conditioned place avoidance, despite adequate control of ongoing pain by treatment with morphine, thereby mirroring breakthrough pain in the clinical setting [41]. Observation that movement-induced breakthrough pain was abolished by ablation of isolectin B4-binding sensory afferents, but not those expressing TRPV1, implicated input from IB4-binding primary sensory nerve fibers in mediating breakthrough pain [41]. Hence, future research aimed at identifying novel targets specific to this population of sensory nerve fibers, is encouraged to inform novel analgesic drug discovery.

### Summary

Despite decades of research on chronic pain mechanisms, including those that underpin PCIBP, there are no novel analgesic agents for the treatment of cancer-induced bone pain and there are also none on the horizon, despite identification of multiple receptors and ion channels that appear to modulate PCIBP in rodent models. Hence, the pharmacological management of PCIBP continues to be informed by the WHO’s Analgesic Ladder that was first promulgated in 1986. Stigma, opioid phobia by patients and misconceptions by clinicians all contribute to the widespread under treatment of cancer-induced bone pain and this needs to be addressed by educational interventions.

## Radiotherapy and bone-targeting agents for relief of metastatic PCIBP

Treatment with external beam radiotherapy, radioisotopes, and/or targeted therapy, in association with analgesics, have important roles in the management of metastatic PCIBP [30]. Radiotherapy and bone-targeting therapy are palliative in nature, aimed at improving the patient’s QoL [80].

### Alpha-emitting radionuclides

The alpha-emitter, radium-233 (Ra-233) was the first targeted alpha therapy approved by the United States (US) Food and Drug Administration (FDA) [50, 62] and the European Medicines Agency (EMA) [84]. Importantly, Ra-233 has a significant survival benefit, both in overall survival (OS) and in the time to the first symptomatic SRE [84, 95]. Specifically, in the ALSYMPCA Phase 3 clinical trial (NCT00699751), Ra-233 had a survival benefit of 3.6 months in patients with mCRPC relative to placebo-treated patients [81]. Ra-233 also delayed the onset of symptomatic skeletal events (SSEs) and improved QoL compared with placebo when added to best standard of care [73, 81]. Ra-233 was efficacious irrespective of docetaxel use [43]. Ra-233 is recommended in the ESMO guideline for use in men with bone-predominant, symptomatic mCRPC without visceral metastases [81]. Regarding mechanism of action, Ra-233 accumulates selectively in bone, particularly in areas of high bone turnover such as the border zones of bone and bone metastases, by forming complexes with hydroxyapatite, the inorganic bone matrix, and inducing double-stranded DNA breaks in tumour cells, osteoblasts and osteoclasts [84, 94]. Ra-233 has a highly localised effect as the alpha particles penetrate to a depth of < 0.1 mm in soft tissues which minimises toxicity to nearby healthy tissues [84]. AEs include nausea, diarrhoea, vomiting and peripheral oedema and the most common (> 10%) haematological abnormalities are anaemia, lymphocytopenia, leukopenia, thrombocytopenia and neutropenia [50].

#### Ra-223 and pain endpoints

The pain responses in patients with mCRPC and treated with Ra-233 either alone or in combination with other treatments in seven clinical studies published in the past 3-years, are summarized in Table 3.

**Table 3:** Pain responses from clinical trials in patients with metastatic prostate cancer induced bone pain who received radionuclides either alone or in combination with chemotherapy, bone targeted agents, local external beam radiation, exercise-based treatment or systemic therapy comprising chemotherapy, small molecule inhibitors or immunotherapy, and published in the past 7 years

Patients (n); treatment description	Pain endpoint; primary, secondary or both	Pain endpoint criteria	Pain relief outcomes	Prominent adverse events	References
Radionucleotides					
α-emitter (Ra-233)					
Patients with mCRPC (n = 354); Ra-223 given up to 6 injections (PARABO study; NCT02398526; a prospective, observational, non-interventional, single-arm study in German real-life nuclear medicine settings)	Primary	Self-reported pain using BPI-SF with ≥ 2 points improvement for ‘worst pain’ in patients with baseline worst pain ≥ 1 point	Of 216 patients who met the 1° endpoint, 59% had ≥ 2 points ↓ in ‘worst pain’ after Ra-223. For those taking and not taking opioids, 65 & 54% met the 1° endpoint respectively. 67%Fof patients who had 5–6 doses of Ra-233, met the 1° endpoint whereas only 43% of those who received ≤ 4 doses met this endpoint	Not reported on	[78]
Patients with mCRPC (n = 29); Phase 2, non-randomized open trial of Ra-233 for pain palliation using standard dosing; Ra-223 at 55 kBq/kg every 4 weeks for 6 doses	Both	1 : 30% ↓ in BPI ‘worst pain’ from baseline to wk 8 that was sustained until wk 12 without escalation of pain medication selected from WHO analgesic ladder 2^0^: BPI pain interference	1° endpoint: met by 31% (9/29) with 21 evaluable patients. For responders, worst pain ↓ 62% & 63% at wk 8 & wk 12 respectively. For all patients, median ↓ in BPI ‘worst pain’ was 11% & 27% at wk 8 & wk 12 respectively 2° endpoints: Pain responders also had median ↓ of 53% in BPI pain interference with both general activity and sleep at wk 12	Not reported on	[63]
Extensively pretreated, symptomatic & asymptomatic patients with progressive mCRPC (n = 300); Ra-223 given at 4 wk intervals (50 or 55 kBq/kg of body weight IV) with number of treatments at physician’s discretion; prospective observational contemporary ‘real-world’ study with patients recruited from 20 Dutch hospitals	Both	1^o^: % patients with a complete pain response (score of 0 on BPI-SF ‘worst pain’) and no ↑ in daily use of analgesics Partial pain response: ≥ 2 points ↓ on BPI-SF ‘worst pain’ or ↓ of ≥ 25% in daily analgesic use. Indeterminate response: ↓in pain not captured by complete or partial responses 2 : % patients with a partial or indeterminant pain response; TPP; PFS; Analgesic use in past 4-wks converted to oral morphine equivalents (mg/day); TTFD; IOCR Questionnaires completed every 4-wks during & after Ra-233 treatment until start of subsequent treatment or death	Of 103 evaluable patients for BPI-SF scores during Ra-233 treatment, 43% had baseline ‘worst pain’ of 5–10 points; 57% had no pain (0–4 scores in BPI-SF) at baseline Complete, partial & indeterminate BPI-SF ‘worst pain’ responses achieved in 31.4%, 26.7%, & 33.3% of patients Median TPP & TTFD were 5.6 & 5.7 mths with no sig. difference between groups that had pain or no pain at baseline respectively Use of analgesics ↓ during Ra-223 treatment & remained low during follow-up. No change in ‘worst pain’ during Ra-223 treatment & follow-up 58% had an IOCR, of whom 49% & 51% had pain & no pain at baseline respectively	Not reported on	[5]
Patients with mCRPC (n = 391); randomised (1:1:1) in open-label Phase 2 trial to 1 of 3 dosing regimens of Ra-223, viz standard dose (55 kBq/kg every 4 wks for 6 cycles) vs high dose (88 kBq/kg every for wks for 6 cycles) vs extended schedule (55 kBq/kg every 4 wks for 12 cycles)	Secondary	Time to pain progression & pain improvement rate using worst pain score on BPI-SF	Standard-dose arm: 27% had pain improvement; High-dose arm: 26% had pain improvement; Extended-schedule arm: 37% had pain improvement Median time to pain progression: Standard-, high-, & extended dose arms = 8.9, 9.4 & 10.1 mths respectively Pain improvement rate: Standard-, high-, & extended dose arms = 27%, 26% & 37% respectively	Fatigue, anaemia, nausea, decreased appetite, bone fractures. 10%, 20% & 19% of patients discontinued study drug in standard, high dose & extended schedule groups respectively, due to adverse events	[92]
Ra-233 plus chemotherapy					
Patients with bone-mCRPC and no visceral metastases (n = 36); 6 standard doses of Ra-223 as either 1st line, 2nd line or 3rd line treatment; retrospective audit	Primary	Pain scores using a numeric rating scale (mean change from baseline to cycle 6)	Sig. ↓ in pain scores from baseline in 2nd and 3rd line groups	22.2% (8/36) reported an AE; anaemia (16.7%), diarrhoea, death	[101]
Chemotherapy-naïve patients with progressive mCRPC & ≥ 2 bone metastases (n = 806); Phase 3 efficacy & safety trial ((NCT02043678, ERA 233) undertaken at 165 oncology & urology centres in 19 countries. Patients were randomized to Ra-223 (up to 6 IV doses at 55 kBq/kg) or placebo (1:1) in addition to oral abiraterone (1000 mg) once-daily plus oral prednisone or prednisolone (5 mg twice-daily) (AAP). Ra-223 or placebo was given at 4 wk intervals	Secondary	Time to pain assessed using the BPI-SF, time to opioid use for cancer pain	At baseline, 49% patients were asymptomatic for pain & 45% had mild pain Time to opioid use: 19.0 mths in AAP plus Ra-233 group relative to 22.6 mths in AAP plus placebo group	Hypertension, urinary tract infection, back pain, pneumonia and traumatic fracture, increased amino alanine transferase activity. 2 treatment-related deaths (1 myocardial infarction & 1 interstitial lung disease) in Ra-233 group & 1 in placebo group (arrhythymia)	[91]
Patients with mCRPC (n = 39); Prospective Phase 2 trial (NCT02507570) of once-daily enzalutamide (160 mg once-daily) combined with Ra-223 (50 kBq/kg) given every 4 wks for up to 6 cycles	Secondary	Bone pain assessed using FACT-P and BPI-SF respectively	Pain ↓ by ≥ 1.0 on BPI-SF composite pain severity score from cycle 1 to cycles 3 to 6 but benefit was lost by end of treatment. No consistent reductions in BPI interference or % pain relief reported by subjects	Fatigue, nausea, anaemia, foot gangrene with sepsis, disease progression leading to death	[90]
β-emitters					
Patients with CRPC and osteoblastic metastases (n = 88); phase 2 Taxium II trial; patients randomised to 1st-line docetaxel infusion every 3 weeks for 10 cycles plus prednisone with or without 2 injections of rhenium-188-HDEP after 3rd & 6th cycles of docetaxel	Secondary	Pain response assessed using a VAS	In both groups, median VAS scores at start of chemotherapy were < 4. After 3 cycles of docetaxel, median VAS-scores were 1 in control group and 2 in rhenium-188 group. No sig. differences in VAS-scores between the two groups	Fatigue & musculoskeletal pain (88% in standard group, 68% in experimental group), neutropenic fever, thrombocytopenia (0% in standard group, 25% in experimental group)	[97]
Patients with PSMA-positive mCRPC previously treated with ≥ 1 androgen-receptor–pathway inhibitor and one or two taxane regimens (n = 831); In Phase 3 VISION open-label, randomized, international trial (NCT04689828), patients randomized 2:1 to Lu-177-PSMA-617 plus protocol permitted standard of care, or standard of care alone respectively. Lu-177-PSMA-617 group received IV infusions of 7.4 GBq every 6 wks for up to 6 cycles	Secondary	Pain assessed using BPI-SF	Time to deterioration of BPI-SF favoured Lu-177-PSMA-617 group relative to control group. Specifically, the median times to worsening pain of ≥ 30% or ≥ 2 points on BPI-SF were 5.9 and 2.2 mths for Lu-177-PSMA-617 and standard of care alone groups, respectively	Fatigue, dry mouth, nausea, pancytopenia, bone marrow failure, subdural haematoma and intracranial haemorrhage	[88]
Bone-targeted agents (with or without a radionuclide and/or docetaxel)					
Patients with mCRPC (n = 757); TRAPEZE (NCT01057810) study with patients randomised to one of 4 arms; docetaxel plus zoledronic acid, docetaxel with Sr-89, docetaxel with zoledronic acid and Sr-89, docetaxel alone	Primary	1°: Clinical PFS. A pain progression–free interval was defined as time in whole days from randomization to date of clinician-determined pain progression	No significant difference in pain progression free interval between the 4 treatment arms	Pain, fatigue, anaemia, febrile neutropenia and neutrophil changes	[47]
Patients with mCRPC with bone metastases (n = 176) of whom > 75% had bone pain; Anti-resorptive therapy (denosumab, pamidronate, zoledronic acid) in real-world PROBone registry study (NCT02410044) in 65 outpatient-centres specialized in medical oncology in Germany	Primary	PROBone questionnaires (FACT-BP, VAS, 1 general pain item, 2 items on taking of medication & effect of medication, 4 time-stress items on daily routine, social life, ability to work & time burden) completed monthly for 12 mths. VAS scores of 0, 1–7 and 9–10 were categorized as ‘no pain’, ‘tolerable pain’ and ‘heavy pain’ respectively	At baseline, 77.2% of patients had pain (VAS), of whom 15% rated pain as ‘heavy’. For patients with ‘heavy’ bone pain, FACT–BP scores improved over time Pain medication was used by 42–52% of patients and its effectiveness was reported as ‘somewhat—quite a bit). This remained stable during the 12-mth study	Renal toxicity, osteonecrosis of the jaw	[45]
Patients with mCRPC (n = 421) who received zoledronic acid, clodronate, or etidonate compared with no treatment/placebo; a network meta-analysis of 21 trials in a Cochrane systematic review	Primary	1: Proportion of patients with pain response measured at baseline, 6 mths, 1 yr, 2 yrs or longest reported follow-up Present Pain Intensity (PPI) score of 0 or ↓ of 2 points without an ↑ in analgesic score or evidence of disease progression, ↓ in analgesic score without an ↑ in PPI score; numeric & linear scales and VAS were used	Little to no difference in pain response for bisphosphonates compared to placebo/no treatment None of the trials reported results for comparison with denosumab	Renal events, osteonecrosis of the jaw	[46]
Local external beam radiation					
Patients with bone-predominant mCRPC with painful bone metastases (n = 6099) received local external beam radiation as a single 8 Gy) or multiple fractions; a systematic review of 26 randomized trials	Primary	Overall response rate of pain evoked by single versus multiple fractions of external beam radiation	Overall pain responses: 70–80% of assessable patients for both single & multiple fraction arms had favourable responses Complete pain responses: ~ 20% of assessable patients for single & multiple fraction arms	Bone pain flare and nausea	[87]
Exercise-based treatment					
Patients with mCRPC on ADT (n = 25); Randomized (1:1:1) to remotely monitored aerobic or resistance exercise or usual care, over a 12-wk period in the pilot CHAMP study (NCT02613273). Aerobic exercise was 20–30 min of moderate-to-vigorous intensity exercise 3–5 days/week. Resistance exercise progressed from 1 to 4 sets of 4–15 repetitions as prescribed by an exercise physiologist	Secondary	Bone pain by VAS assessment at each exercise session; number of participants using opioid medication for pain between baseline & 12 weeks	Patients in aerobic (n = 8) and resistance (n = 7) groups reported no perceived bone pain (median VAS score = 0/10)	Study-related joint or bone pain (n = 3/25)	[50]
Systemic therapy (chemotherapy, small molecule inhibitors, immunotherapy)					
Patients with mCRPC with symptomatic bone metastases on bone scan and bone pain that required opioid analgesic treatment (n = 117); double-blind, randomized phase 3 trial (COMET-2, NCT01522443); patients randomized to oral cabozantinib (tyrosine kinase inhibitor) at 60 mg once-daily plus placebo infusion (≤ 10 infusions) plus oral prednisone-matched placebo twice-daily every 3-wks, or mitoxantrone at 12 mg/m^2^ every 3 weeks (≤ 10 infusions) plus twice-daily oral prednisone at 5 mg plus an oral cabozantinib-matched placebo once-daily	Primary	Rate of pain response at week 6 & confirmed at week 12, defined as a ≥ 30% ↓ from baseline in mean daily ‘worst pain’ score using a minimum of four BPI-SF reports during a 7-day period without an increase in daily opioid use, use of a new opioid analgesic type or clinical pain progression	1° endpoint was not met as rates of pain response were 15% & 17% for cabozantinib & mitoxantrone-prednisone respectively. This 2% difference was not significant	Fatigue, decreased appetite, nausea, diarrhoea, anaemia and hypertension	[8]
Patients with asymptomatic or minimally symptomatic chemotherapy-naïve, mCRPC without visceral metastases whose disease had progressed during hormonal therapy and discontinuation of prior ADT (n = 602); double-blind phase 3 trial (NCT01057810) where patients randomized (2:1) to IV ipilimumab at 10 mg/kg or placebo every 3 wks for up to 4 doses. followed by double-blind maintenance treatment with ipilimumab or placebo every 12 wks	Secondary	PFS & TPP using BPI-SF Pain intensity assessed every 12-wks by daily patient report using an Analgesic Use Diary & BPI-SF for 5 consecutive days at each assessment point TPP defined as time to ↑ in mean daily ‘worst pain’ of ≥ 2 points from baseline, maintained over two consecutive periods, initiation of opioid analgesics or palliative radiotherapy or an ↑ from baseline in mean analgesic score ≥ 25%	Number of patients with a pain response was too small to evaluate potential treatment-related differences	Diarrhoea, rash, fatigue, pruritis, nausea, decreased appetite and vomiting, death	[11]
Patients with chemotherapy-naïve mCRPC (n = 1168); Open-label, randomized phase 3 trial (FIRSTANA; NCT01308567) at 159 centres across 25 countries; most patients had previously received hormonal therapy; patients randomized to either IV C20, or C25 or D75 ((1:1:1) every 3 weeks plus oral prednisone at 10 mg once-daily Patients underwent a median of 9-treatment cycles C20 = cabazitaxel 20 mg/m^2^ C25 = cabazitaxel 25 mg/m^2^ D75 – docetaxel 75 mg/m^2^	Secondary	Pain PFS, pain response, QOL (FACT-P) Pain response assessed using McGill-Melzack PPI scale	Median time to pain PFS was 8.0, 7.3, and 10.1 mths for C20, C25, and D75, respectively with no sig. difference between treatment groups, although pain PFS was numerically improved with D75 relative to C25 % patients with pain response did not differ sig. between treatment groups (C20: 45.5%, C25: 43.3%, D75: 42.7%) % patients with pain progression did not differ sig. between treatment groups (C20: 59.1%, C25: 61.6%; D75: 54.0%,)	Febrile neutropenia, neutropenic infection, diarrhoea, haematuria, peripheral neuropathy, stomatitis, peripheral oedema, alopecia, & nail disorders	[77]
Patients with mCRPC who had disease progression within 12 mths of receiving either abiraterone or enzalutamide (n = 255) were recruited into clinical trial #NCT02485691; patients were randomized (1:1) to either IV cabazitaxel every 3 wks at 25 mg/m^2^ with once-daily prednisone and G-CSF (7 cycles), or the other androgen-signaling-targeted inhibitor (abiraterone 1000 mg plus prednisone daily or enzalutamide 160 mg once-daily) for 4-cycles	Secondary	Pain progression by BPI-SF or level of WHO cancer pain analgesia used WHO cancer pain analgesic level of 2 to 3 [on a 3-point scale, with higher numbers indicating use of stronger analgesic agents	At baseline, 69% of patients had pain progression Pain response was evaluable in 111 patients in cabazitaxel group & 109 patients in androgen-signalling-targeted inhibitor group A confirmed pain response was achieved in 45% of patients in the cabazitaxel group, but only 19.3% in the androgen-signalling-targeted inhibitor group	Infection, bronchial aspiration, functional deterioration due to disease progression, spinal cord compression and head injury, pulmonary thromboembolism, cardiac disorder, cerebral bleeding, renal failure and general health deterioration	[28]
Patients with mCRPC (n = 150); ProCAID phase 2 trial (NCT02121639) with patients randomly assigned to either oral capivasertib (320 mg twice-daily, 4 days on/3 days off from day 2 each cycle) or matched placebo in combination with IV docetaxel (75 mg/m^2^, day 1 each cycle) & twice-daily prednisone (5 mg) for up to 10 cycles at 3 wk intervals. Study continued until disease progression	Secondary	Bone pain changes using the BPI	No sig. differences in bone pain between treatment arms up to treatment cycle 5	Diarrhoea, fatigue, nausea	[26]
Patients with metastatic castration sensitive prostate cancer with at least 1 distant bony metastasis & baseline pain & who had had a minimum of 2-wks and up to 6 previous cycles of docetaxel and ADT for ≤ 6-mths (n = 609); Phase 3 double-blind, placebo-controlled TITAN trial (NCT02489318) conducted at 260 sites across 23 countries, with patients randomized (1:1) to ADT plus either oral apalutamide (240 mg/day) or matched placebo Median follow up times were similar between treatment groups at 19 & 22 mths	Secondary	Pain progression & time to chronic opioid use. Pain responses self-assessed using BPI-SF with improvement as ≥ 2 points	Apalutamide group: 29% and 22% sig. more likely to have improvement in ‘worst pain’ and ‘average pain’ than for placebo group Risk of deterioration of some BPI-SF pain items (pain interference with mood, pain interference with sleep, pain interference score) was sig. lower for apalutamide than for placebo group Mean TTD was sig. longer in apalutamide than placebo group (28.7 vs 21.8 mths respectively) as well as for pain at its least in last 24 h & for interference with mood, walking ability, relations, and sleep	None reported	[3]

For three of four studies where Ra-233 was given alone, pain responses were the primary endpoint (Table 3). In the PARABO (NCT02398526) study, ~ 60% of patients had a ≥ 2-point decrease in ‘worst pain’ on the Brief Pain Inventory-Short Form (BPI-SF), irrespective of whether or not they were taking opioids [78]. In a small open-label Phase 2 trial of Ra-233 for pain palliation in patients with mCRPC (Table 3), 31% had a decrease in ‘worst pain’ of ≥ 30% from baseline to week 8, which was sustained until week 12 without escalation of pain medication [63]. Pain responders also had a median 53% decrease in pain interference with both general activity and sleep at week 12 (Table 3) [63].

Comparison of high-dose and extended-dosing schedules of Ra-233 in an open label, randomised trial, relative to standard dosing, for treating mCRPC [92] showed that for ‘worst pain’ on the BPI-SF, the pain improvement rates were 27%, 26% & 37% for standard-, high- and extended-dose arms respectively but for the latter this was offset by a higher rate of intolerable side-effects (Table 3).

In a phase 3 trial (NCT02043678), Ra-223 or placebo was combined with oral abiraterone acetate plus oral prednisone or prednisolone (AAP), in patients with chemotherapy-naïve, progressive, mCRPC with ≥ 2 bone metastases (Table 3) [91]. The median time to opioid use was shorter at 19 months for the AAP plus Ra-233 group, *c.f.* ~ 23 months for the AAP plus placebo group [91]. The less favourable pain response for the combination treatment group was aligned with worse safety outcomes for the same patients (Table 3) [91]. This trial was unblinded prematurely after more fractures and deaths were observed in the Ra-233 group than the placebo group [91]. Overall, addition of Ra-223 to AAP did not improve SSE-free survival and it was associated with increased frequency of bone fractures (29%) compared with placebo (11%) (Table 3) [91]. These findings led to revision of prescribing recommendations for Ra-223 by the FDA and the EMA [91]. A small prospective Phase 2 trial (NCT02507570) of Ra-223 or placebo combined with oral enzalutamide in patients with mCRPC showed that initial benefit on pain responses was lost by the end of treatment [90]. There were no significant safety signals or AEs to contraindicate the combined use of enzalutamide and Ra-233 [90], in contrast to combined dosing of Ra-233 with AAP (Table 3) [91].

#### Beta-emitting radionuclides

Advantages of beta-emitters including strontium-89 (^89^Sr), samarium-153 (^153^Sm), lexidronam (^153^Sm-EDTMP), phosphorus-32 (^32^P) sodium phosphate and rhenium-188 (^188^Re) are their ability to treat multiple disease sites simultaneously, ease of administration, repeatability and potential integration with other treatments [40]. However, in contrast to the α-emitter, Ra-233, these agents do not extend overall survival in patients with mCRPC [82]. However, in March 2022, this situation changed when the FDA approved lutetium (Lu)-177-prostate specific membrane antigen (PSMA)-617 for the treatment of patients with PSMA-positive mCRPC who had been treated previously with an androgen receptor pathway inhibitor and taxane-based chemotherapy [2]. FDA approval was granted based upon the data from the Phase 3 VISION clinical trial (NCT03511664).

#### β-emitters and pain endpoints

In the open-label Phase 3 VISION trial, PSMA-positive patients with mCRPC and randomized to receive Lu-177-PSMA-617 plus standard of care, had superior pain responses *c.f.* patients given standard of care alone (Table 3) [88]. Specifically, the median time to worsening pain of ≥ 30% or ≥ 2-points on the BPI-SF, was longer at 5.9 months for the Lu-177-PSMA-617 group *c.f.* 2.2 months for the standard of care alone group (Table 3) [88]. In other work, patients with mCRPC were randomized to treatment with 10 cycles of docetaxel plus prednisone every 3 weeks with or without rhenium-188-HDEP treatment after the 3rd and 6th cycles of docetaxel (Table 3). The VAS pain scores decreased to 1 and 2 in the control and Re-188 groups respectively, showing similar benefit on pain outcomes between the two groups (Table 3) [97].

### Bone-targeting agents and pain endpoints

Clinical practice guidelines, including the ESMO-endorsed Cancer Care Ontario guideline or the American Society of Clinical Oncology (ASCO) guideline, recommend starting bone-targeted agents (BTA) such as bisphosphonates (zoledronic acid (ZA), pamidronate) or the RANK inhibitor (denosumab) for treating patients with newly diagnosed mCRPC, irrespective of whether or not they are symptomatic [24, 45, 88, 99]. The aim is to prevent SREs and pain progression, as well as stabilization of patient’s QoL (Table 3) [45]. Clinical trial comparison of 4- versus 12-weekly BTA regimens in patients with bone metastases due to mCRPC, showed equivalence for both efficacy and health-related QoL [23, 42]. Thus, de-escalation of denosumab, ZA and pamidronate treatment from 4- to 12-weekly is a reasonable treatment option in these patients [23]. Since widespread adoption of bisphosphonates and denosumab in the routine care of patients with mCRPC, the incidence of SREs has fallen significantly in non-clinical trial populations [99].

In the TRAPEZE study (NCT01057810) (Table 3), pain progression free intervals did not differ significantly in patients with mCRPC randomized to docetaxel plus ZA, docetaxel plus Sr-89, docetaxel plus ZA plus Sr-89 or docetaxel alone, showing no additional benefit on pain responses *c.f.* that produced by docetaxel alone [47]. In the PROBone study (NCT02410044) (Table 3), 77% of patients with bone metastases due to mCRPC had bone pain at baseline, 15% of whom rated their pain as ‘heavy’ which equated to a VAS score of 9–10 [45]. Once taking a BTA, patients with ‘heavy’ bone pain improved over the 12 month study duration (Table 3) [45]. Additionally, in a network meta-analysis of 21 clinical studies in men with mCRPC and bone metastases, there was little to no difference in pain responses for bisphosphates *c.f.* placebo/no treatment, and none of the trials reported pain response results for denosumab (Table 3) [46]. Thus, future trials are needed to provide data on pain responses evoked by denosumab in men with mCRPC.

### Local EBRT and pain endpoints

In a systematic review of 26 randomized trials in patients with painful uncomplicated bone metastases due to mCRPC (Table 3), the analgesic efficacy of a single fraction of EBRT did not differ significantly from that of multiple fractions in patients [87]. Overall, 40% of responders achieved pain relief within 10 days of a single fraction and one third of patients achieved complete pain relief (Table 3) [24].

### Exercise-based treatment and pain endpoints

A pilot trial (CHAMP study; NCT02613273) in men with mCRPC who were receiving androgen deprivation therapy (ADT), showed that aerobic and resistance exercise interventions prescribed by an exercise physiologist and remotely monitored, were well tolerated over a 12 week period (Table 3) [50]. Also, patients reported a median overall bone pain VAS score of zero during the 12 week study (Table 3) [50]. The successful tailoring of an exercise program that avoided patients’ metastatic sites to prevent injury, is encouraging [50].

### Systemic therapy (chemotherapy, small molecule inhibitors and immunotherapy)

In the past 5 years, six studies reported on pain responses in patients with PCIBP who received either chemotherapy, small molecule inhibitors, or immunotherapy. However, pain response was a primary outcome in only one of these studies (Table 3). Disappointingly, in this latter randomized phase 3 trial (COMET-2; NCT01522443), the primary pain endpoint was not met irrespective of whether patients received either the tyrosine kinase inhibitor, cabozantinib plus placebo infusions every 3 weeks plus oral prednisone-matched placebo twice-daily, or mitoxantrone every 3 weeks plus twice-daily prednisone plus a once-daily oral cabozantinib-matched placebo (Table 3) [8].

In a phase 3 trial (NCT01057810), asymptomatic/minimally symptomatic, chemotherapy-naïve patients with mCRPC without visceral metastases, and whose disease had progressed during hormonal treatment, and ADT had been discontinued, were recruited and randomized to receive ipilimumab or placebo every 3 weeks for up to 4 doses (Table 3). However, the number of patients with a pain response was too small to evaluate possible treatment-related differences [11]. In the FIRSTANA phase 3 trial (NCT01308567), patients who had chemotherapy-naïve mCRPC, were randomized to cabazitaxel at 20 or 25 mg/m^2^ (C20 and C25 respectively) or docetaxel at 75 mg/m^2^ (D75), plus prednisone once-daily, for a median of 9 treatment cycles [77]. Overall, the median times to pain progression-free survival, the percentage of patients with a pain response and those with pain progression did not differ significantly between the three treatment arms (Table 3) [77]. In other work, patients with mCRPC disease progression within 12-months of having received abiraterone or enzalutamide, were randomized (NCT02485691) to cabazitaxel every 3-weeks with once-daily prednisone and granulocyte colony-stimulating factor, or to the other androgen-signaling-targeted inhibitor once-daily [28]. Encouragingly, more than twice as many patients had a confirmed pain response in the cabazitaxel group (45%) *c.f.* the androgen-signalling-targeted inhibitor group (19%) (Table 3) [28]. Disappointingly, in the ProCAID phase 2 trial (NCT02121639) in patients with mCRPC who received either the pan-AKT inhibitor, capivasertib, or matched placebo in combination with docetaxel & twice-daily prednisone for up to 10 cycles at 3-week intervals, there were no significant differences in bone pain between the two treatment regimens up to treatment cycle 5 (Table 3) [26]. In the TITAN phase 3 trial (NCT02489310), patients with mCRPC who received continuous ADT, and the anti-androgen, apalutamide, had consistently favourable pain scores and a significantly longer time to deterioration of pain compared with those given ADT and placebo, which is encouraging (Table 2) [3].

In summary, from the foregoing, administration of a radionuclide either alone or in combination with chemotherapy, to patients with mCRPC improved pain responses in many patients (Table 3). Additionally, a systematic review of 26 clinical studies showed that a single fraction of local EBRT was as effective as multiple fractions (Table 3). The improved pain responses achieved in a pilot study in patients with mCRPC prescribed aerobic or resistance exercise an exercise-physiologist (Table 3), warrant follow-up with a larger trial. For patients with PCIBP treated with chemotherapy, small molecule inhibitors and/or immunotherapy agents, there were variable pain responses (Table 3). As pain response was the primary endpoint in only one of these six trials (Table 3), future work addressing this gap in knowledge on pain outcomes, is warranted.

## Conclusion

In men with mCRPC, the prevalence of bone metastases is 90% and median survival time is 12–53 months. Bone metastases may result in SREs and debilitating bone pain that greatly impair the QoL of patients. Current treatments for alleviating PCIBP include radiotherapy, bone-targeting therapy, and analgesic/adjuvant agents that are mainly palliative in nature, aimed at improving pain relief and patient QoL. Recent clinical trials show that radionuclides, given either alone or in combination with chemotherapy, improved pain responses in many patients and a systematic review showed unequivocally that a single fraction of local EBRT was as effective as multiple fractions. However, treatment with chemotherapy, small molecule inhibitors and/or immunotherapy agents, did not improve pain responses in the majority of trials (Table 3). Disappointingly, there are no novel analgesic agents on the horizon for the relief of PCIBP and this is an area of large unmet medical need that warrants concerted research attention.
